# Neurocognitive disorders: what are the prioritized caregiver needs? A consensus obtained by the Delphi method

**DOI:** 10.1186/s12913-018-3826-y

**Published:** 2018-12-29

**Authors:** Teddy Novais, Christelle Mouchoux, Michel Kossovsky, Lucie Winterstein, Floriane Delphin-Combe, Pierre Krolak-Salmon, V. Dauphinot

**Affiliations:** 1 EA-7425 HESPER, Health Services and Performance Research, University Lyon, F-69003 Lyon, France; 2 Pharmaceutical Unit, Charpennes Hospital, Hospices Civils de Lyon, F-69100 Lyon, France; 3 University Lyon 1, F-69000 Lyon, France; 4 INSERM U1028, CNRS UMR5292, Lyon Neuroscience Research Center, Brain Dynamics and Cognition Team, F-69000 Lyon, France; 5 Department of Internal medicine, Rehabilitation and Geriatrics, University Hospitals of Geneva and University of Geneva, Geneva, Switzerland; 6 Clinical and Research Memory Centre of Lyon (CMRR), Charpennes Hospital, Hospices Civils de Lyon, F-69100 Lyon, France

**Keywords:** Caregivers, Neurocognitive disorders, Delphi method, Needs assessment, Training/education

## Abstract

**Background:**

The symptoms related to neurocognitive disorders (NCD) may lead to caregiver burden increase. Involving caregivers in research may be an effective way of improving the practicalities and relevance of interventions. The aim of this study was to gather opinion and gain consensus on the caregivers ‘priorities, using a Delphi method and including aspects of needs in pharmaceutical dimension.

**Methods:**

Observational study using a modified Delphi method. This study was conducted in the Clinical and Research Memory Center of the University Hospital of Lyon (France), between September 2015 and January 2016. The expert panel was composed of 68 informal caregivers of people with subjective cognitive decline or NCD living at home.

**Results:**

Caregivers assigned a very high importance to the dimension “information needs about their relative’s disease”, i.e. *information on the disease, the treatment and the research*; and to “coping skills”, i.e. skills related to *emotional support, communication, relationship evolution with the relative and skills to cope with behavioural crisis, behavioural and cognitive disorders*. The aspect “*coping with behavioural disorders*” received a high selection rate (83%).

**Conclusions:**

The main needs selected can be used to design relevant interventions and give guidance to policy to support caregivers. To meet caregiver’s needs, interventions should focus on information about disease and treatment and psychoeducational interventions.

## Background

Cognitive impairment, functional autonomy loss and behavioural disorders caused by neurocognitive disorders (NCD) as Alzheimer’s Disease and Related Diseases (ADRD) may lead to caregiver burden increase [1, 2]. The informal caregiver, i.e. not professional, is often a spouse or a child, providing assistance with daily living activities, managing the relative’s behavioural symptoms, coordinating supportive services, facilitating healthcare visits and making financial and healthcare decisions [3]. They play a central role in the care of people with NCD given caregiver informal care account for more than half of societal costs [4] However, the burden of care may have physical, psychological, emotional, social and financial consequences on caregiver and increase caregiver frailty may cause early patient institutionalization [5, 6]. In addition, previous studies have shown a large number of unmet needs were associated with a higher burden and an increase in caregiver strain and depressive symptoms [3, 7–9]. In NCD, the number of unmet needs is high while the levels of services utilization are low [10]. Is there a discrepancy between the services and support proposed to caregivers and the services that they need?

Involving informal caregivers to understand their needs from their own perspectives may be an effective way of improving the practicalities, acceptability and relevance of services and support [11].

Several studies using quantitative or qualitative approaches have evaluated the caregivers’ needs in terms of information, skills, support and services in medical and psychosocial dimensions [12, 13]. However, it appears that the needs assessment instruments used in these studies did not explore all dimensions of needs. This is the case for the needs in pharmaceutical dimension while people with NCD and their caregivers represent populations with higher risk of developing drug-related problems due to aging, comorbidities associated with polypharmacy and caregiver neglect of their own health care [14–19]. Previous studies have identified an amount of needs in informal caregivers but no study has hierarchized their main needs in medical, pharmaceutical and psychosocial dimensions. Identifying the main caregivers’needs and hierarchizing them would allow to design targeted and appropriate interventions to meet the needs of the greatest number. The aim of this study was to gather opinion and gain consensus on the caregivers ‘priorities, using a Delphi method and including aspects of needs in pharmaceutical dimension. The intended outcome was to identify the main common needs among informal caregivers of people with NCD.

## Methods

### Study design

A two round modified Delphi process was used to generate consensus amongst a panel of informal caregivers. The Delphi method was chosen because this method allows to define a consensus in panel of experts on a specific topic from a large number of predefined items with a technique for the indirect confrontation of opinions [20]. This method also allows to hierarchize the main items retained round after round by the experts. The Delphi process uses a series of questionnaires to collect information from participants in a number of rounds. In the case of modified Delphi, the starting point is a pre-derived list of questions established by analysts [21]. A decision was made to restrict the Delphi process to two rounds before inviting experts to participate, since the initial questionnaire was based upon a careful review of available literature. It was predicted that two rounds would be enough to reach adequate consensus and would minimise the burden and the difficulty of the process for the caregivers of people with NCD.

### Setting and participants

Informal caregivers, considered as experts of this Delphi survey, were recruited at the Clinical and Research Memory Center of the University Hospital of Lyon (Hospices Civils de Lyon, France), between the September 1, 2015 and January 30, 2016. Inclusion criteria were: informal caregivers of people living at home with NCD and agreeing to participate to the Delphi survey [22]. The informal caregiver was defined as a nonprofessional person living with their relative or providing support to their relative.

### Detailed Delphi study scheme

#### Development of the survey questionnaire

The first step was to develop the survey questionnaire for the first round. The aspects of needs in the survey were based on a systematic review whose purpose was to review the methodologies used to identify the needs, the existing needs assessment instruments and the main topics of needs explored among caregivers of patients with mild cognitive impairment to dementia [12]. The selection criteria were: studies using quantitative, qualitative and mixed method to assess the caregiver’s needs in terms of information, coping skills, support and service. Several aspects of needs related to pharmaceutical care have been added to the questionnaire. The first draft of the questionnaire was developed by the first author and was independently reviewed by analysts specialized in the care of ADRD patients to establish content validity. The group of analysts included 2 geriatricians, a neuro-geriatrician, 2 neuropsychologists, 2 pharmacists, a social worker and a methodologist. This allowed the identification of 46 aspects of needs into 3 dimensions: (i) 19 aspects in the dimension of information needs (INF), (ii) 18 aspects in the dimension of skills needs (SKI) and (iii) 9 aspects in the dimension of support needs (SUP). Fifteen of the 46 aspects of needs involved the pharmaceutical field, including information about caregiver’s and patient’s drugs and medication management.

#### Round 1 method

First, an information letter explaining the study and the procedure for the first round was given to the caregivers after a memory consultation of their relatives. Second, the first questionnaire was distributed to caregivers who agreed to participate.

The first round consisted of rating the pre-selected aspects of needs. Caregivers were asked to rate the importance of each of these 46 aspects on a nine-point scale with three anchors: 1 “not important”; 5 “of moderate importance”; 9 “extremely important”. Respondents were also informed that they could add other aspects of needs.

The second round was based on a second questionnaire drawn up from the responses obtained at the first round, using two criteria to determine the retained aspects: they had to be both “consensual” and “important”, i.e. to have obtained 75% of the answers in one of the three parts of the scale [1–9] and a median score ≥ 8. This threshold has been shown to favour high reliability [23].

#### Round 2 method

The second questionnaire of the Delphi process was sent to the home of the same panel of experts for the second round. In each round, stamped envelopes were provided to the informal caregivers to send back the questionnaire.

The second round aimed to select the main aspects of needs according to the same panel of experts, i.e. aspect of needs selected by 50% and over of caregivers (representing the majority of caregivers). Amongst the proposed aspects in the second questionnaire, caregivers were asked to select the ten aspects that they considered to be “the most important” and to rank them in order of importance from 1 to 10, with 10 being the most important and 1 the least important. The rank given by the caregiver for each aspect of the second questionnaire was equated to a weight of importance (e.g. the rank 10 equals a weight of 10).

The second round analysis was based on the number of experts who selected each aspect of needs and the total weight of importance (sum of the weights granted by caregivers for each aspect). The more an aspect of needs was selected by the experts and more its total weight was important, the more this aspect was important for the caregivers.

In the second round, questions about the caregivers ‘preferences regarding the implementation of identified needs in information, skills and support were also added in another questionnaire. The preferences in terms of intervention timing (i.e. on the demand, one shot at the diagnosis or several times all along the disease) and means (i.e. leaflet, internet, individual or collective session, hotline) were investigated.

### Data collection

Data collected at the inclusion included: sociodemographic characteristics (age, gender, educational level), relationship with the relative, the length of caregiving, the caregiver burden using the short version of Zarit Burden Interview (ZBI) and patient medical data: diagnosis, global cognitive function using the Mini Mental State Examination (MMSE), behaviour using the Neuropsychiatric Inventory (NPI), the functional autonomy level using the Instrumental Activities of Daily Living (IADL). The short version of ZBI score ranged from 0 (no burden) to 7 (higher burden) [5, 24]. The French version of MMSE was previously validated for the detection of cognitive impairment using the DSM-III criteria [25]. The IADL assessed 8 instrumental activities and the score ranging from 0 (dependent) to 8 (independent) [26]. The NPI evaluates 10 behavioral domains and 2 neurovegetative troubles [27]. A higher overall NPI score (maximum 144) indicates more severe behavioral disorders. All data were entered in an electronic database to perform the analyses.

### Data analysis

Relative with NCD and caregivers characteristics were described at baseline with means ± standard deviations (SD) or frequencies (percentages). The participation rate to the second round of the survey was reported and characteristics of caregivers who completed the two round and those who achieved only the first round were compared. Normally distributed data were compared using the student’s t test and paired categorical variables using Chi-square test. For the round 1, the results were described using means ± SD and median of the rating assigned on the scale to each aspect of needs. The response rates in the three parts of the scale [1–9] was also described for each aspect of needs. For the round 2, the frequency of selection and the weight of each aspect of needs from the second questionnaire were detailed. The weight of an aspect of needs corresponds to the sum of the rank (10 to 1) granted by the caregivers. Data were analysed using the Excel software (Microsoft Office Excel 2010) and SPSS (Statistical Package for the Social Sciences) version 21.0 for Windows (SPSS Inc., Chicago, Illinois, USA).

### Ethical consideration

The observational study protocol has been reviewed and approved by a local ethics committee the 8/7/2015 (Comité de Protection des Personnes Sud-Est IV, references L15–119).

## Results

### Panel of experts

Between September 2015 and January 2016, sixty eight caregivers of people with NCD participated in the first round of the survey. Fifty eight caregivers (85%) participated to the second round. There were no significant differences between the groups that participated in only round 1 and completed in both. The characteristics of the caregivers and their relatives were summarized in Table 1.

**Table 1:** Experts i.e. informal caregivers’ characteristics and people with NCD characteristics

	*n = 68*
Gender - n (%)	
female	46 (67.5%)
Relationship with their relative- n (%)	
spouses	43 (63.0%)
children	20 (29.5%)
other	5 (7.5%)
Age – mean ± SD	69.1 ± 12.0
age of spouses – mean ± SD	75.3 ± 8.5
age of children – mean ± SD	56.0 ± 7.1
age ≥ 65 – n (%)	42 (62%)
Educational level - n (%)	
nil	5 (7.5%)
primary	9 (13.0%)
secondary	33 (48.5%)
tertiary	21 (31.0%)
Cohabitation with their relative - n (%)	
yes	49 (72.0%)
Length of caregiving - n (%)	
< 1 year	12 (17.5%)
1–5 year(s)	48 (70.5)
> 5 years	8 (12.0%)
Caregiver Burden (short ZBI) – mean ± SD	3.7 ± 1.9
People with NCD characteristics	
Age – mean ± SD	80.0 ± 8.0
Gender - n (%)	
Female	35 (51.5%)
Measurements - mean ± SD	
Mini-Mental State Examination	17.4 ± 6.2
Neuropsychiatric Inventory	21.8 ± 14.8
Instrumental Activities of Daily Living	3.0 ± 2.8
Disability Assessment of Dementia DAD-16	5.3 ± 5.8
Diagnosis stage - n (%)	
Subjective cognitive decline	4 (6.0%)
Minor neurocognitive disorders	15 (22.0%)
Major neurocognitive disorders	49 (72.0%)
Diagnosis etiology - n (%)	
Alzheimer’s Disease	38 (56.0%)
Alzheimer’s Disease with cerebrovascular disease	12 (17.5%)
Lewy body disease	4 (6.0%)
Cerebrovascular disease	5 (7.0%)
Other neurocognitive disorders	9 (13.5%)

*ZBI* Zarit Burden Index

### Round 1

The mean score of importance of the 46 aspects was 6.93 ± 2.49, varying according to the dimensions of needs (Table 2). The SUP dimension was the least important, with a mean score of 4.98 ± 2.94. In this dimension, the caregivers attributed the lowest scores to the pharmaceutical support. They attributed a mean score of 7.33 ± 2.07 for the INF dimension and 7.46 ± 2.17 for the SKI dimension. In these dimensions, the highest scores were attributed to the needs of *information about their relative’s disease* and *providing affective support to their relative*. Twenty aspects of needs met the first decision-rule made to draw up the second questionnaire. No aspect of needs in support has been selected for the second round.

**Table 2:** First round - rating of the 46 needs aspects (n = 68)

				Distribution of the ratings along the scale (%)		
Needs aspects	DIM^a^	Median	Mean (SD)	1–3	4–6	7–9
Information on the neurocognitive disease and its progression^b^	INF-P	9	8.39 (1.10)	0	7	93
Providing emotional and affective support to PWNCD^b^	SKI-C	9	8.32 (1.32)	2	6	92
Information on cognitive disorders^b^	INF-P	9	8.23 (1.13)	0	9	91
The diagnostic process of the neurocognitive disease^b^	INF-P	9	8.19 (1.24)	0	11	89
Communication between caregivers and PWNCD^b^	SKI-C	9	8.17 (1.41)	0	14	86
Management of the PWNCD pain^b^	SKI-C	9	8.17 (1.35)	2	6	92
Coping with cognitive disorders^b^	SKI-C	9	8.11 (1.39)	0	15	85
Coping with behavioral crisis at home^b^	SKI-C	9	8.09 (1.66)	2	12	86
Coping with behavioral disorders^b^	SKI-C	9	8.01 (1.49)	1	12	87
The research (disease and therapies) ^b^	INF-P	9	8.01 (1.34)	0	16	84
Roles of the health professionals involved in the PWNCD care pathway^b^	INF-P	9	7.96 (1.39)	0	15	85
Information on behavioral disorders^b^	INF-P	9	7.94 (1.74)	5	10	85
Information on the drugs for the neurocognitive disease (efficacy, tolerance and utilization) ^b^	INF-P	9	7.84 (1.69)	4	14	82
Undertaking the steps for the services utilization ^b^	SKI-S	9	7.50 (2.35)	9	13	78
Finding available services closer to home^b^	SKI-S	9	7.49 (2.31)	7	15	78
Learning to adjust the sedative drug dose based on the patient’s condition (e.g. sedation, agitation)	SKI-M	8.5	6.97 (2.7)	14	19	67
Coping with the relationship evolution between caregivers and PWNCD^b^	SKI-C	8	7.83 (1.40)	0	20	80
Stimulating/appropriates activities for PWNCD at home^b^	SKI-C	8	7.79 (1.59)	1	19	80
Basic care to PWNCD^b^	SKI-C	8	7.74 (1.67)	2	15	83
Information on the PWNCD drugs for comorbidities (efficacy, tolerance and utilization) ^b^	INF-P	8	7.56 (1.66)	3	21	76
Coping with feeling related to caring (e.g, frustration, culpability, stress)	SKI-C	8	7.53 (1.79)	3	23	74
Financial issues for the ADL^b^	INF-S	8	7.5 (2.19)	8	13	79
Information on non-pharmacological treatment for PWNCD	INF-P	8	7.45 (1.59)	0	30	70
Communicating on the PWNCD disease and the caregiver role with the entourage	SKI-C	8	7.44 (1.85)	3	26	71
Information on nursing care at home	INF-S	8	7.24 (2.41)	9	17	74
Legal issues	INF-S	8	6.98 (2.24)	7	26	67
Information on respite, day care	INF-S	8	6.90 (2.65)	13	17	70
Information on institutionalization and long-term care unit	INF-S	8	6.90 (2.50)	11	25	64
Being able to manage the PWNCD drugs	SKI-M	8	6.55 (2.87)	20	20	61
Information on the consequences of caregiving (caregiver health)	INF-C	7	7.09 (1.97)	5	29	66
Learning to seek and accept help from the entourage	SKI-C	7	6.92 (2.01)	3	35	62
Information on associations and foundations	INF-S	7	6.60 1.93)	5	38	57
Information on environmental safety	INF-S	7	6.34 2.37)	12	33	55
Coping with difficulties to administer the PWNCD drugs	SKI-M	7	6.22 (2.82)	20	25	55
Review of the PWNCD drug prescribing by a pharmacist (optimization of the therapeutics)	SUP-P	7	6.01 (2.68)	18	31	51
Being able to manage their drugs (caregiver’s drugs)	SKI-M	7	5.40 (3.33)	35	14	51
Annual medical check-up for caregiver by a GP	SUP	6.5	6.23 (2.70)	17	33	50
Information on drugs for caregiver’s psychological disorders	INF-C	6	6.25 (3.35)	10	45	45
Information on the caregiver’s drugs (efficacy, tolerance and utilization)	INF-C	6	5.98 (2.58)	16	42	42
Support by a social worker	SUP	5	5.67 (2.92)	24	36	40
Obtaining a form with timing of PWNCD drug administration	SUP-P	5	5.62 (2.82)	20	39	41
Identification and resolution of difficulties associated with the PWNCD drug management	SUP-P	5	5.44 (2.77)	22	39	29
Emotional support for the caregiver by a psychologist	SUP	5	4.76 (2.56)	29	44	27
Review of the caregiver drug prescribing by a pharmacist (optimization of the therapeutics)	SUP-P	2.5	3.62 (2.81)	53	28	19
Identification and resolution of the difficulties associated with the caregiver’s drug management	SUP-P	2.5	3.52 (2.70)	53	28	19
Obtaining a form with timing of caregiver drug administration	SUP-P	2	3.42 (2.90)	58	23	19

*ADL* activities of the daily living, *GP* general practitioner, *PWNCD* people with a neurocognitve dirsorder

^a^Dimension of need. *INF-P* information needs about patient (people with NCD), *INF-C* information needs about caregiving, *INF-S* information needs about services, *SKI-C* coping skills needs, *SKI-M* Skills needs about medication, *SKI-S* skills needs about services, *SUP* support needs for the caregiver, *SUP-P* pharmaceutical support

^b^Aspect of need selected for the second round

Several aspects of needs were assessed as important but not consensual (dissensus): 9 areas of needs with a median score ≥ 8 were assessed as “extremely important” by 61 to 74% of the caregivers in the round 1. This notably concerned skills *to cope with feeling related to caring*, *to communicate on the relatives’ disease,* and *caregiving with their entourage*; as well as information needs about *non-pharmacological treatment* and *nursing care at home*. No other aspect of needs was added by the respondents for the second round after the questionnaires analysis.

### Round 2

The questionnaire of the second round was composed of 20 aspects of needs evaluated as “consensual” and “important” by the expert panel. Ten needs aspects (top ten list) were selected by 50% and over caregivers (50 to 83%). Amongst the ten most selected needs aspects, four pertained to the dimension “information needs about the relative’s disease”, i.e. *information on the disease, the treatment and the research*; and six to “coping skills”, i.e. skills related to *emotional support, communication, relationship evolution with the relative and skills to cope with behavioural crisis, behavioural and cognitive disorders* (Table 3). The aspect “*coping with behavioural disorders*” received a high selection rate (83%). However, seven aspects of needs among the ten remaining aspects were also reported to be of importance for more than one third of the caregivers.

**Table 3:** Second round - selecting of the 20 needs aspects (*n* = 58)

Aspect of needs	DIM^a^	Selection	Percent	Weight	Rank
		Number of experts			
Coping with behavioral disorders	SKI-C	48	83	307	1
Coping with behavioral crisis	SKI-C	41	71	280	2
Information on the neurocognitive disease and its progression	INF-P	37	64	240	3
Information on behavioral disorders	INF-P	38	66	219	4
Providing emotional and affective support to PWNCD	SKI-C	35	60	214	5
Communication between caregivers and PWNCD	SKI-C	28	48	177	6
Coping with cognitive disorders	SKI-C	28	48	175	7
The research (disease and therapies)	INF-P	37	64	170	8
Coping with the relationship evolution between caregivers and PWNCD	SKI-C	30	52	152	9
Information on the drugs for the neurocognitive disease (efficacy, tolerance and utilization)	INF-P	29	50	147	10
Information on cognitive disorders	INF-P	23	40	146	–
Stimulating/appropriates activities for PWNCD at home	SKI-C	25	43	133	–
Management of the PWNCD pain	SKI-C	21	36	128	
Roles of the health professionals involved in the PWNCD care pathway	INF-P	22	38	111	–
Finding available services closer to home	SKI-S	28	48	100	–
Undertaking the steps for the services utilization	SKI-S	26	45	100	–
Financial issues for the ADL	INF-S	23	40	94	–
The diagnostic process of the neurocognitive disease	INF-P	18	31	92	–
Basic care to PWNCD	SKI-C	15	26	85	–
Information on the PWNCD drugs for comorbidities (efficacy, tolerance and utilization)	INF-P	11	19	41	–

*ADL* activities of the daily living, *PWNCD* people with a neurocognitve dirsorder

^a^Dimension of need. *INF-P* information needs about patient (people with NCD); *INF-S* information needs about services, *SKI-C* coping skills needs, *SKI-S* skills needs about services

### Focus on pharmaceutical care needs

Amongst the fifteen aspects of needs in pharmaceutical dimension in the round 1, three aspects were ranked as important and consensual by the caregivers such as *research* (therapies and disease), *information about patient’s drugs for the cognitive disease* and *information about patient’s drugs for comorbidities*. However, two others aspects were ranked as important but not consensual (dissensus): *adjustment of sedative drug dosage* and the *management of the patient’s drug*. The aspects of needs about informal caregivers’ medication were the lowest ranked. In the round 2, two aspects of needs in pharmaceutical dimension were included in the top ten list (selected by 50% and over of caregivers): *research* and *information about patient’s drugs for the cognitive disease* (rank 8 and 10 respectively).

### Preferences about information delivery and skill acquisition

Table 4 summarizes the caregivers’ preferences. For the information delivery and the skill acquisition, the majority of caregivers said that they would prefer multiple individual interviews with an appropriate health professional all along the disease progression of their relatives.

**Table 4:** Caregivers’ preferences about information delivery and skills acquisition features

Means of information delivery	%	Means of skills acquisition	%
Timing		Timing	
Several times all along the disease	72	Several times all along the disease	62
One shot, at the diagnosis of the disease	18	One shot, at the diagnosis of the disease	25
On demand	10	On demand	13
Other	0	Other	0
Means		Means	
Personal interview	53	Personal interview	49
Internet site	19	Collective session	30
Leaflet or video	13	Hotline	21
Collective workshop	15	Other	0
Other	0		

## Discussion

The Delphi findings present an informal caregiver consensus on their priority needs in the field of NCD.

Previous studies using other methodologies have evaluated the caregiver’s needs in NCD. A systematic review of 12 qualitative studies has identified two aspects of needs: *management of older people with dementia* and *caregivers’ personal needs* [13]. The first aspect included similar needs to the present study: information and disease knowledge, activities of daily living support, behavioral and psychological symptoms of dementia support and, formal/informal care support. The second aspect included caregivers’ physical and psychological health and management of caregivers’ own lives. *Wackerbarth* et al. have used quantitative method through self-administered needs assessment surveys [28]. Caregivers of individuals with ADRD were asked to rate information and support needs in terms of importance. The most important information needs involved *the health plan coverage* (financial issue), *the means to find the best care* and *information about diagnosis and therapeutics*, especially current treatment options, risks and benefits and treatments for behaviors. The most important support needs identified by caregivers were mainly for the relative as *providing emotional support* and *understand the relative’s feelings*. Rather than support for themselves, caregivers rated support for their relatives as most important [28]. Similarly, in the present Delphi study, informal caregivers have given low rank to the domains of needs about their own concerns such as *emotional support for the caregiver* and *annual medical check-up by general practitioner for the caregiver.* Caregivers often appear to ignore their own personal and health needs to concentrate on providing the best care possible to the ill person [29–32]. This is more noticeable with medication-related needs, as caregivers did not perceive needs for their own medication management, at least not as much as for their relatives. Older people themselves, informal caregivers are also exposed to polypharmacy with a higher risk of developing drug-related problems. *Thorpe* et al. have shown that 33% of patients living with dementia and 39% of their caregivers were taking at least one potentially inappropriate medication [18]. These aspects of pharmaceutical needs mainly met the needs of health professionals while informal caregivers did not perceive possible benefits.

The Delphi process is rarely used in non-professional subjects, especially in informal caregivers to establish a consensus. *Bond* et al. developed a set of guidelines on how family and non-professional helpers may assist an older person who is developing neurocognitive impairment, or has dementia or delirium [31]. They used two expert panels: health professional panel and caregiver advocate panel (people who were members of the national Alzheimer’s organization or a caregiver organization and held a leadership position within an advocacy organization). Family or informal caregivers were not included in the building of these guidelines. Another study using the Delphi process in dementia was conducted with professionals and informal caregivers of people living with dementia to identify the main resilience factors i.e. personal strategies helping to face difficulties related to the disease [32]. The items *patients’ behavioral problems* and *feeling competent as a caregiver* were selected as essential resilience features. Caregivers also emphasized the importance of *social supports*, the *quality of the relationship with their relative,* while professionals considered *coping skills*, and *a good quality of life of caregivers* most relevant. These studies indicated that priorities of caregiver advocates/informal caregivers and professionals differed to some extent [11, 32].

Dickinson et al. and Gilhooly et al. showed in their Meta-analyzes that the most effective psychosocial interventions for caregivers were multicomponent and psychoeducational interventions [33, 34]. In the meta-analysis of Sörensen et al., psychoeducational interventions were defined as interventions that “involve a structured program geared toward providing information about the care receiver’s disease process and about resources and services and training caregivers to respond effectively to disease-related problems, such as memory and behavior problems in dementia patients” [35]. In this present study, ten common caregiver’s needs are identified allowing to design a common core of educational and psychoeducational interventions. To meet their needs, these interventions should be focused on (1) information about the disease, the behavioral disorders, the medication and research; (2) coping skills regarding behavioral and cognitive disorders, behavioral crisis at home, relationship changes; and (3) skills to communicate and to provide affective support to their relatives. In addition, specific needs, i.e. important but not consensual needs (dissensus), have been also highlighted in the first round.

A multicomponent intervention integrating personalized pharmaceutical care to a multidisciplinary psychosocial program for caregivers of people with ADRD (PHARMAID study protocol) was designed and is being evaluated [36]. This collaborative program aims to optimize the medication management of the patient with ADRD and their caregiver, to provide appropriate information and to develop coping skills based on collective and individual interventions. The finding of this study will assess the effectiveness of this collaborative approach.

### Strengths and limitations

To our knowledge, this is the first study reporting prioritized informal caregivers’ needs in NCD by using a Delphi process. A main strength of the present study is the involvement of caregivers in a Delphi survey to be experts of their situation and to identify their proper needs. Using a consensus of people with real-life experience allows highlighting information and suggestions that may be relevant and useful to caregivers dealing with cognitive and behavioral disorders. The use of a ranking process in the second round enables caregivers to provide a clear prioritization of their needs. Another advantage of the Delphi methodology is the possibility to obtain anonymously the views of a diverse group of persons, preventing the influence of dominant individuals [32, 37]. The present study has been conducted in a single-centre specialized in the care of people with cognitive complaints in France and with a mostly urban population of caregivers. Despite the high number of recruited experts as compared to other Delphi studies [38, 39], the present study identified needs that may not be internationally extendable. Indeed, a large variability in care structures and organizations in the medico-social field is observed across countries, as well as discrepancy in health care accessibility between urban and rural caregivers. The first round questionnaire of the Delphi method was developed form a systematic review of quantitative and qualitative studies and proposed a wide range of needs. However, even if the group of analysts who validated this questionnaire had a global vision on health and social-care provision, it did not include all professionals involved in the care of people with NCD (eg. nurses, occupational therapists). Moreover, the questionnaire of the first round was distributed after the consultation with the specialist. The rating could be affected by the service and content of memory consultation (e.g. if the caregiver felt that memory consultation could not provide adequate information on the disease of cognitive disorder, the subject may rate that it was very important to provide information on cognitive disorder). A measure of the caregiver satisfaction or dis-satisfaction about the memory consultation could have prevented this limitation. Finally, caregivers’ needs may be subject to factors, such as relationship to their relative, disease stage, caregiving length and others. The sample size of caregivers did not allow to achieve subgroup analysis.

## Conclusion

The main needs selected from informal caregivers perspectives can be used to design relevant intervention studies and give guidance to policy to support caregivers of people with NCD more effectively and better tailored to their needs. To meet the informal caregiver’s needs and to reduce their burden, interventions focused on disease and treatment information and psychoeducational interventions should be prioritized. In order to provide personalized support to informal caregivers, this study should be replicated internationally in rural and urban caregivers and individual needs should be assessed ahead of the educational process.

## Abbreviations

ADRD: Alzheimer’s disease and Related Disorders
INF: Information needs
NCD: Neurocognitive Disorders
SD: Standard Deviations
SKI: Skills needs
SUP: Support needs
